# Two new calcicolous caloplacoid lichens from South Korea, with a taxonomic key to the species of *Huriella* and *Squamulea*

**DOI:** 10.3897/mycokeys.84.71227

**Published:** 2021-11-01

**Authors:** Beeyoung Gun Lee, Jae-Seoun Hur

**Affiliations:** 1 Baekdudaegan National Arboretum, Bonghwa 36209, Republic of Korea Baekdudaegan National Arboretum Bonghwa Republic of Korea; 2 Korean Lichen Research Institute, Sunchon National University, Suncheon 57922, Republic of Korea Sunchon National University Suncheon Republic of Korea

**Keywords:** Biodiversity, phylogeny, saxicolous, taxonomy, Teloschistaceae

## Abstract

*Pyrenodesmiarugosa* Lee & Hur and *Huriellaaeruginosa* Lee & Hur are described as new lichen-forming fungi from a calcareous mountain of South Korea. *Pyrenodesmiarugosa* is distinguishable from *Pyrenodesmiamicromontana* (Frolov, Wilk & Vondrák) Hafellner & Türk, the most similar species, by thicker thallus, rugose areoles, larger apothecia, shorter hymenium, shorter hypothecium and narrower tip cells of paraphyses. *Huriellaaeruginosa*, the second new species, differs from ‘Squamulea’ chelonia Bungartz & Søchting by dark greenish-grey to grey thallus without pruina, gold to yellow-brown epihymenium, larger ascospores and thallus K– and KC– reaction. Molecular analyses employing internal transcribed spacer (ITS), mitochondrial small subunit (mtSSU) and nuclear large subunit ribosomal RNA (LSU) sequences strongly support the two caloplacoid species to be distinct in their genera. A surrogate key is provided to assist in the identification of all 20 taxa in *Huriella* and *Squamulea*.

## Introduction

Many lichens are only detected in calcareous areas, particularly for crustaceous lichens, as many plants are never found, except on calcareous rocks and soils (Watson 1918; Kossowska 2008; Pykälä et al. 2017). Caloplacoid lichens have been discovered in calcareous areas, such as *Pyrenodesmiaalbopustulata* (Khodos. & S.Y. Kondr.) I.V. Frolov & Vondrák, *P.badioreagens* (Tretiach & Muggia) Søchting, Arup & Frödén, *P.concreticola* (Vondrák & Khodos.) Søchting, Arup & Frödén, *P.erodens* (Tretiach, Pinna & Grube) Søchting, Arup & Frödén, ‘Squamulea’ chelonia, *Squamuleagalactophylla* (Tuck.) Arup, Søchting & Frödén,‘Squamulea’ humboldtiana Bungartz & Søchting, *Squamuleaparviloba* (Wetmore) Arup, Søchting & Frödén and *S.subsoluta* (Nyl.) Arup, Søchting & Frödén (Khodosovtsev et al. 2002; Tretiach et al. 2003; Wetmore 2003; Tretiach and Muggia 2006; Vondrák 2008; Arup 2013; Bungartz et al. 2020). Many lichens have been introduced from the calcareous areas in Korea, such as *Anemadecipiens* (A. Massal.) Forssell, *Astroplacaloekoesiana* S.Y. Kondr., Farkas, J.J. Woo & Hur, *Caeruleumheppii* (Nägeli ex Körb.) K. Knudsen & Arcadia, *Clauzadeametzleri* (Körb.) Clauzade & Cl. Roux, *Clauzadeamonticola* (Ach.) Hafellner & Bellem., *Collemaauriforme* (With.) Coppins & J.R. Laundon, *C.cristatum* (L.) Weber ex F.H. Wigg., *Endocarponpallidum* Ach., *Halecaniapakistanica* van den Boom & Elix, *Heppiaadglutinata* A. Massal., *Ionaspisepulotica* (Ach.) Blomb. & Forssell, *Lecaniaturicensis* (Hepp) Müll. Arg., *Lecanoraalbescens* (Hoffm.) Branth & Rostr., *L.semipallida* H. Magn., *Lemmopsisarnoldiana* (Hepp) Zahlbr., *Lichinellacribellifera* (Nyl.) P.P. Moreno & Egea, *L.stipatula* Nyl., *Placynthiumtantaleum* (Hepp) Hue, *Porinafluminea* P.M. McCarthy & P.N. Johnson, *Psorotichiafrustulosa* Anzi, *P.schaereri* (A. Massal.) Arnold, *Pterygiopsisaffinis* (A. Massal.) Henssen, Pyrenocarponaff.thelostomum (Ach. ex J. Harriman) Coppins & Aptroot, *Rufoplacaaesanensis* S.Y. Kondr. & Hur, *Staurothelefrustulenta* Vain., *Synalissaramulosa* (Hoffm.) Körb., *Thyreaconfusa* Henssen, *Toniniapoeltiana* S.Y. Kondr., Lőkös & Hur, *T.tristis* (Th. Fr.) Th. Fr. and *Verrucariamuralis* Ach. (van den Boom and Elix 2005; Joshi et al. 2009; Schultz and Moon 2011; Aptroot and Moon 2014, 2015, Kondratyuk et al. 2016a, 2016b, 2017a, 2020). Although calcicolous caloplacoid lichens were little reported from Korea in the past, for example, *Rufoplacaaesanensis*, it is assumed that diverse caloplacoid lichens inhabit calcareous rocks and soils which were previously reported from just rock or soil without specifying specific rock or soil types.

**Figure 1.**
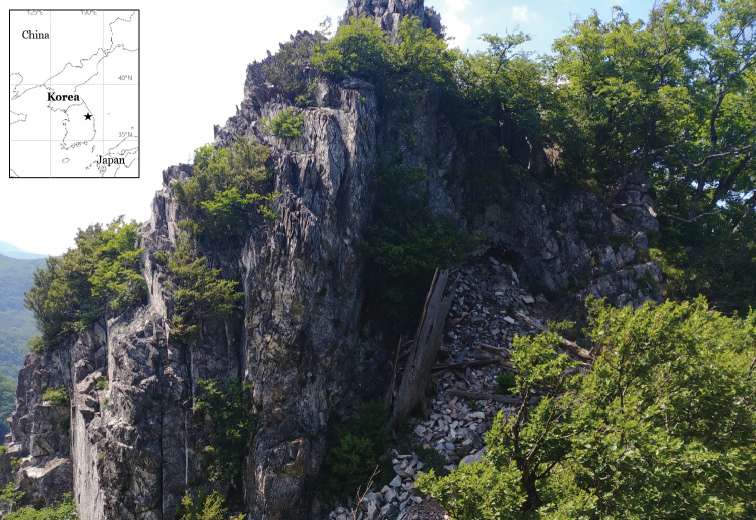
Specific collection site for two new species, representing the habitat/landscape and the location (black star mark).

This study describes two new calcicolous caloplacoid lichens in the genera *Pyrenodesmia* and *Huriella*. Qualified field surveys for the lichen diversity on the Baekdudaegan Mountains, the main mountain range stretching across the entire Korean Peninsula, were accomplished during the summer of 2020 and a few dozen specimens of caloplacoid lichens were collected in Mt. Seokbyung, a calcareous mountain (Fig. 1). We describe them as two new species, *Pyrenodesmiarugosa* and *Huriellaaeruginosa*. The specimens are deposited in the herbarium of the Baekdudaegan National Arboretum (KBA), South Korea.

## Materials and methods

### Morphological and chemical analyses

Hand-cut sections were prepared with a razor blade under a stereomicroscope (Olympus optical SZ51; Olympus, Tokyo, Japan), examined under a compound microscope (Nikon Eclipse E400; Nikon, Tokyo, Japan) and imaged using a software programme (NIS-Elements D; Nikon, Tokyo, Japan) and a DS-Fi3 camera (Nikon, Tokyo, Japan), mounted on a Nikon Eclipse Ni-U microscope (Nikon, Tokyo, Japan). The ascospores were investigated at 1000× magnification in water. The length and width of the ascospores were measured and the range of spore sizes was shown with average, standard deviation and number of measured spores. Thin-layer chromatography (TLC) was performed using solvent systems A and C according to standard methods (Orange et al. 2001).

### Isolation, DNA extraction, amplification and sequencing

Hand-cut sections of ascomata or thallus from all collected specimens were prepared for DNA isolation and DNA was extracted with a NucleoSpin Plant II Kit in line with the manufacturer’s instructions (Macherey-Nagel, Düren, Germany). PCR amplification for the internal transcribed spacer region (ITS1-5.8S-ITS2 rDNA), the mitochondrial small subunit and the nuclear large subunit ribosomal RNA genes was achieved using Bioneer’s AccuPower PCR Premix (Bioneer, Daejeon, Korea) in 20-μl tubes and primers ITS5 and ITS4 (White et al. 1990), mrSSU1 and mrSSU3R (Zoller et al. 1999) and LR0R and LR5 (Rehner and Samuels 1994), respectively. The PCR thermal cycling parameters used were 95 °C (15 sec), followed by 35 cycles of 95 °C (45 sec), 54 °C (45 sec) and 72 °C (1 min) and a final extension at 72 °C (7 min), based on Ekman (2001). DNA sequences were generated by the genomic research company Macrogen (Seoul, Korea).

### Phylogenetic analyses

All ITS, mtSSU and LSU sequences were aligned and edited manually using ClustalW in Bioedit V.7.2.6.1 (Hall 1999). All missing and ambiguously aligned data and parsimony-uninformative positions were removed and only parsimony-informative regions were finally analysed in MEGA X (Stecher et al. 2020). The final alignment comprised 878 (ITS), 900 (mtSSU) and 1701 (LSU) columns for *Pyrenodesmia*. In them, variable regions were 178 (ITS), 42 (mtSSU) and 618 (LSU). The phylogenetically-informative regions were 356 (ITS), 55 (mtSSU) and 98 (LSU). The final alignment for *Huriella* and *Squamulea* comprised 693 (ITS) columns. In them, variable regions were 78 (ITS). Finally, the phylogenetically-informative region was 246 (ITS). Phylogenetic trees with bootstrap values were obtained in RAxML GUI 2.0 beta (Edler et al. 2019) using the Maximum Likelihood method with a rapid bootstrap with 1000 bootstrap replications and GTR GAMMA for the substitution matrix. The posterior probabilities were obtained in BEAST 2.6.4 (Bouckaert et al. 2019) using the HKY (Hasegawa, Kishino and Yano) model, as the appropriate model for nucleotide substitution, based on the Bayesian Information Criterion (BIC) (Schwarz 1978) as evaluated by bModelTest (Bouckaert and Drummond 2017), empirical base frequencies, gamma for the site heterogeneity model, four categories for gamma and a 10,000,000 Markov Chain Monte Carlo chain length with a 10,000-echo state screening and 1000 log parameters. Then, a consensus tree was constructed in TreeAnnotator 2.6.4 (Bouckaert et al. 2019) with a burn-in of 5000, no posterior probability limit, a maximum clade credibility tree for the target tree type and median node heights. All trees were displayed in FigTree 1.4.2 (Rambaut 2014) and edited in Microsoft Paint. The bootstrapping and Bayesian analyses were repeated three times for the result consistency and no significant differences were shown for the tree shapes and branch values. The phylogenetic trees and DNA sequence alignments are deposited in TreeBASE under the study ID 28190.

## Results and discussion

### Phylogenetic analyses

**Figure 2.**
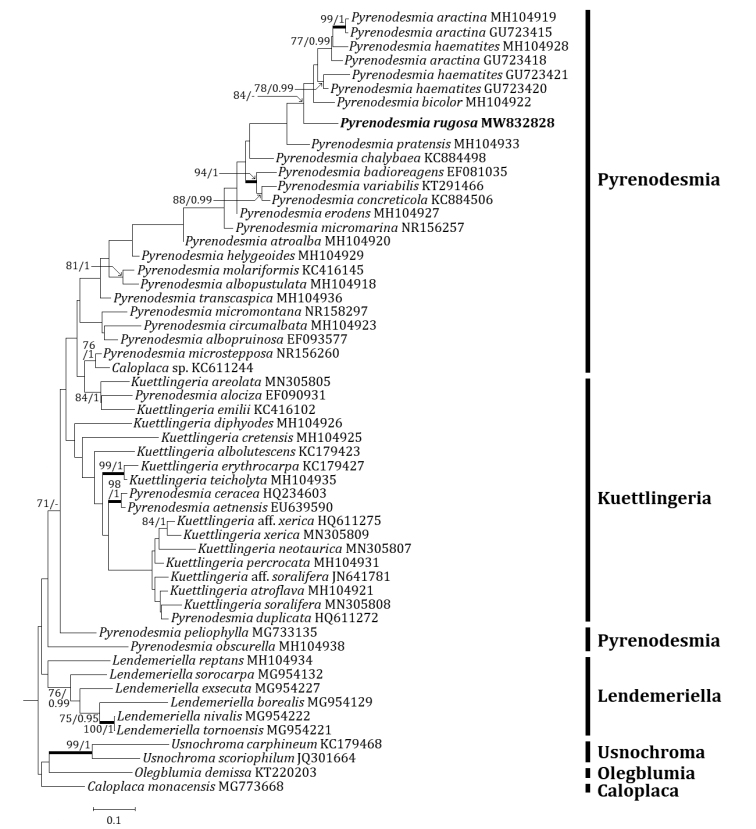
Phylogenetic relationships amongst available species in the genus *Pyrenodesmia*, based on a Maximum Likelihood analysis of the dataset of ITS sequences. The tree was rooted with the sequences of the genera *Caloplaca*, *Lendemeriella*, *Olegblumia* and *Usnochroma*. Maximum Likelihood bootstrap values ≥ 70% and posterior probabilities ≥ 95% are shown above internal branches. Branches with bootstrap values ≥ 90% are shown in bold. The new species *Pyrenodesmiarugosa* is presented in bold and all species names are followed by the GenBank accession numbers. Reference Table 1 provides the species related to the specific GenBank accession numbers and voucher information.

**Figure 3.**
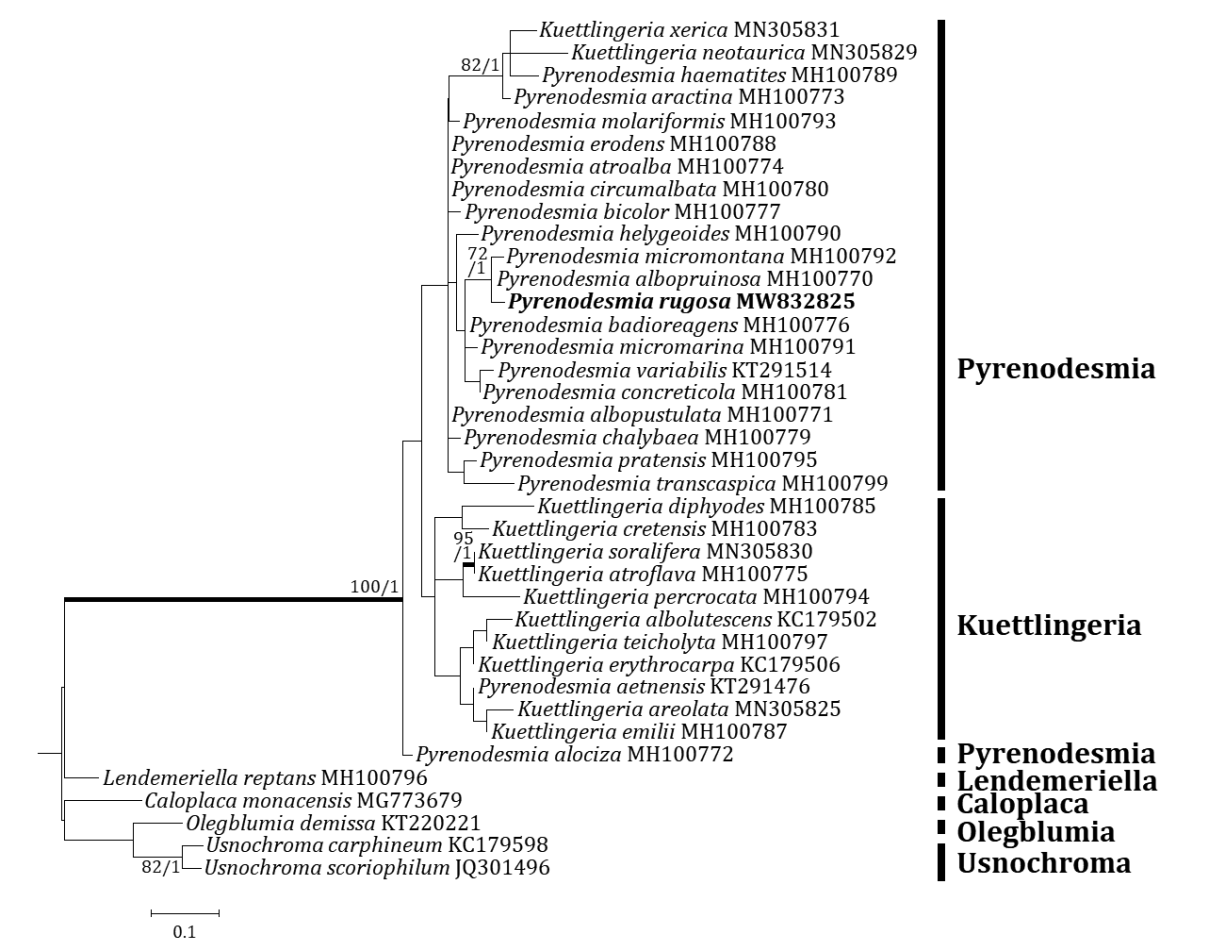
Phylogenetic relationships amongst available species in the genus *Pyrenodesmia*, based on a Maximum Likelihood analysis of the dataset of the mitochondrial small subunit (mtSSU) sequences. The tree was rooted with five sequences of the genera *Caloplaca*, *Lendemeriella*, *Olegblumia* and *Usnochroma*. Maximum Likelihood bootstrap values ≥ 70% and posterior probabilities ≥ 95% are shown above internal branches. Branches with bootstrap values ≥ 90% are shown in bold. The new species *Pyrenodesmiarugosa* is presented in bold and all species names are followed by the GenBank accession numbers. Reference Table 1 provides the species related to the specific GenBank accession numbers and voucher information.

**Figure 4.**
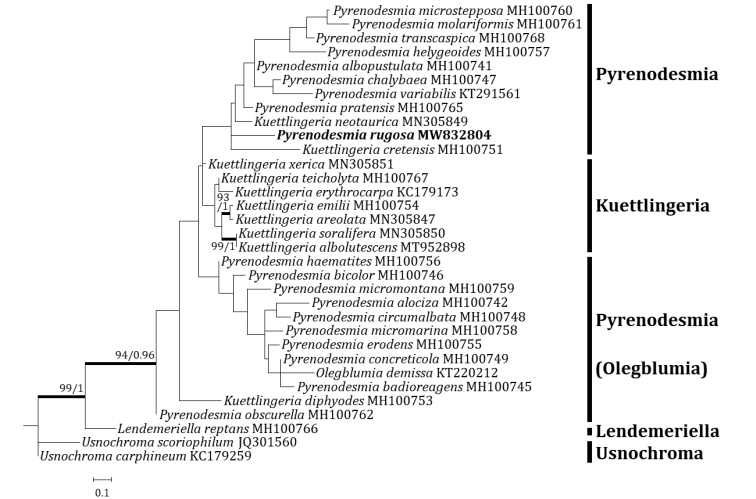
Phylogenetic relationships amongst available species in the genus *Pyrenodesmia* , based on a Maximum Likelihood analysis of the dataset of the nuclear large subunit ribosomal RNA (LSU) sequences. The tree was rooted with three sequences of the genera *Lendemeriella* and *Usnochroma*. Maximum Likelihood bootstrap values ≥ 70% and posterior probabilities ≥ 95% are shown above internal branches. Branches with bootstrap values ≥ 90% are shown in bold. The new species *Pyrenodesmiarugosa* is presented in bold and all species names are followed by the GenBank accession numbers. Reference Table 1 provides the species related to the specific GenBank accession numbers and voucher information.

**Figure 5.**
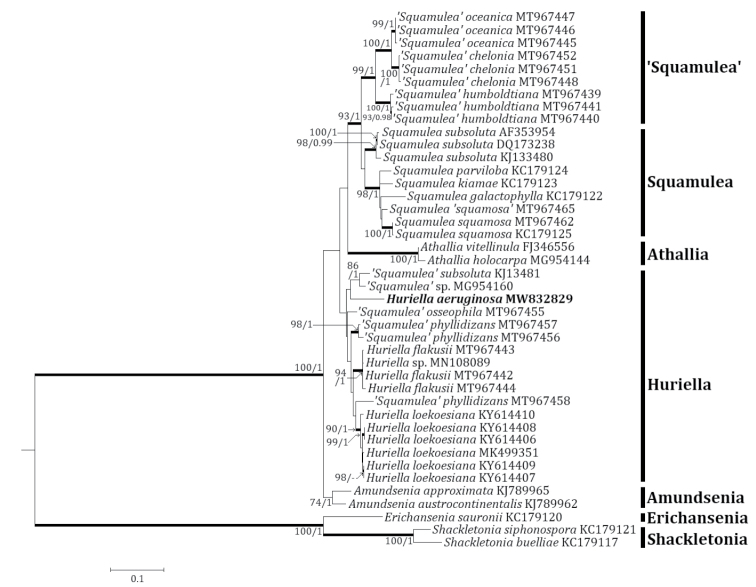
Phylogenetic relationships amongst available species in the genera *Huriella* and *Squamulea*, based on a Maximum Likelihood analysis of the dataset of ITS sequences. The tree was rooted with the sequences of the genera *Amundsenia*, *Erichansenia* and *Shackletonia*. Maximum Likelihood bootstrap values ≥ 70% and posterior probabilities ≥ 95% are shown above internal branches. Branches with bootstrap values ≥ 90% are shown in bold. The new species *Huriellaaeruginosa* is presented in bold and all species names are followed by the GenBank accession numbers. Reference Table 1 provides the species related to the specific GenBank accession numbers and voucher information.

Three independent phylogenetic trees for *Pyrenodesmia* and one independent phylogenetic tree for *Squamulea* were produced from 165 sequences (96 for ITS, 37 for mtSSU and 32 for LSU) from GenBank and four new sequences (two for ITS, one for mtSSU and one for LSU) for the new species (Table 1). *Pyrenodesmiarugosa*, a new species, was positioned in the genus *Pyrenodesmia* in all ITS, mtSSU and LSU trees. The ITS tree described that the new species was solely located without any clade. Several species closely positioned with the new species were *Pyrenodesmiaaractina* (Fr.) S.Y. Kondr., *P.bicolor* (H. Magn.) S.Y. Kondr. and *P.haematites* (Chaub. ex St.-Amans) S.Y. Kondr., represented by a bootstrap value of 84 and a posterior probability of 0.73 (not shown) for the branch (Fig. 2). The mtSSU tree showed that the new species was located in a clade with *Pyrenodesmiaalbopruinosa* (Arnold) S.Y. Kondr. and *P.micromontana*, represented by a bootstrap value of 72 and a posterior probability of 1.0 for the branch (Fig. 3). The LSU tree depicted that the new species was positioned solely without any clade. Several species, such as *Kuettlingeriacretensis* (Zahlbr.) I.V. Frolov & Vondrák, *K.neotaurica* (Vondrák, Khodos., Arup & Søchting) I.V. Frolov, Vondrák & Arup, *Pyrenodesmiaalbopustulata*, *P.chalybaea* (Fr.) A. Massal., *P.helygeoides* (Vain.) Arnold, *P.microstepposa* (Frolov, Nadyeina, Khodos. & Vondrák) Hafellner & Türk, *P.molariformis* (Frolov, Vondrák, Nadyeina & Khodos.) S.Y. Kondr., *P.pratensis* (Wetmore) Frolov & Vondrák and *P.variabilis* (Pers.) A. Massal. are situated close to the new species (Fig. 4). *Huriellaaeruginosa*, the second new species, was located in *Huriella* in the ITS tree. The ITS tree described that the new species was positioned in a clade with ‘Squamulea’ subsoluta and ‘*Squamulea*’ sp., represented by a bootstrap value of 35 (not shown) without a posterior probability as the Maximum Likelihood analysis did not match with the Bayesian Inference for the clade (Fig. 5). Although the two closely located sequences were named for *Squamulea* in the beginning, they are close to *Huriella*, not *Squamulea*. The two sequences are arranged in the genus *Huriella* with the new species. The phylogenetic analyses did not designate any species identical to the two new species in each genus *Pyrenodesmia* and *Huriella*.

**Table 1.** Species list and DNA sequence information employed for phylogenetic analysis.

No	Species	ID (ITS)	ID (mtSSU)	ID (LSU)	Voucher
1	*Amundseniaapproximata*	KJ789965			L08179 (LD)
2	*Amundseniaaustrocontinentalis*	KJ789962			21966 (HO)
3	*Athalliaholocarpa*	MG954144			Vondrak 18072
4	*Athalliavitellinula*	FJ346556			Arup L03052
5	*Caloplacamonacensis*	MG773668	MG773679		Malicek 8255
6	*Caloplaca* sp.	KC611244			CBFS:JV6943
7	*Erichanseniasauronii*	KC179120			Sochting 7654
**8**	***Huriellaaeruginosa***	**MW832829**			**BDNA-L-0001072**
9	*Huriellaflakusii*	MT967442			Bungartz 4131 (CDS 28162)
10	*Huriellaflakusii*	MT967443			Bungartz 4157 (CDS 28188)
11	*Huriellaflakusii*	MT967444			Aptroot 65261 (CDS 31847)
12	*Huriellaloekoesiana*	KY614406			KoLRI 15423
13	*Huriellaloekoesiana*	KY614407			KoLRI 19017
14	*Huriellaloekoesiana*	KY614408			KoLRI 40141
15	*Huriellaloekoesiana*	KY614409			KoLRI 40236
16	*Huriellaloekoesiana*	KY614410			KoLRI 40238
17	*Huriellaloekoesiana*	MK499351			HKAS 102112
18	*Huriella* sp.	MN108089			KRAM-L-70242
19	*Kuettlingeriaalbolutescens*	KC179423	KC179502	MT952898	Arup L09030 (LD)
20	*Kuettlingeriaareolata*	MN305805	MN305825	MN305847	Vondrak 10843
21	*Kuettlingeriaatroflava*	MH104921	MH100775		Vondrak 8723 (PRA)
22	*Kuettlingeriacretensis*	MH104925	MH100783	MH100751	Frolov s.n.
23	*Kuettlingeriadiphyodes*	MH104926	MH100785	MH100753	Frolov 1430
24	*Kuettlingeriaemilii*	KC416102	MH100787	MH100754	JV9358
25	*Kuettlingeriaerythrocarpa*	KC179427	KC179506	KC179173	Arup L07109 (LD)
26	*Kuettlingerianeotaurica*	MN305807	MN305829	MN305849	Vondrak 7213
27	*Kuettlingeriapercrocata*	MH104931	MH100794		Vondrak 4634 (PRA)
28	*Kuettlingeriasoralifera*	MN305808	MN305830	MN305850	Vondrak 10813
29	*Kuettlingeriaaff.soralifera*	JN641781			CBFS:JV8325
30	*Kuettlingeriateicholyta*	MH104935	MH100797	MH100767	Vondrak 6943 (PRA)
31	*Kuettlingeriaxerica*	MN305809	MN305831	MN305851	Vondrak 14544
32	*Kuettlingeriaaff.xerica*	HQ611275			CBFS:JV7618
33	*Lendemeriellaborealis*	MG954129			Vondrak 11073
34	*Lendemeriellaexsecuta*	MG954227			Spribille 24441
35	*Lendemeriellanivalis*	MG954222			Spribille 29306
36	*Lendemeriellareptans*	MH104934	MH100796	MH100766	Lendemer 48186 (NY)
37	*Lendemeriellasorocarpa*	MG954132			Vondrak12695
38	*Lendemeriellatornoensis*	MG954221			Spribille 29473
39	*Olegblumiademissa*	KT220203	KT220221	KT220212	SK C65
40	*Pyrenodesmiaaetnensis*	EU639590	KT291476		X. Llimona (BCN)
41	*Pyrenodesmiaalbopruinosa*	EF093577	MH100770		TSB 37658
42	*Pyrenodesmiaalbopustulata*	MH104918	MH100771	MH100741	Vondrak 10463 (PRA)
43	*Pyrenodesmiaalociza*	EF090931	MH100772	MH100742	TSB 37735
44	*Pyrenodesmiaaractina*	GU723415			Bornholm 5907
45	*Pyrenodesmiaaractina*	GU723418			Bornholm 6911
46	*Pyrenodesmiaaractina*	MH104919	MH100773		Vondrak 6702 (PRA)
47	*Pyrenodesmiaatroalba*	MH104920	MH100774		Spribille s.n.
48	*Pyrenodesmiabadioreagens*	EF081035	MH100776	MH100745	TSB 36422
49	*Pyrenodesmiabicolor*	MH104922	MH100777	MH100746	Vondrak 10373 (PRA)
50	*Pyrenodesmiaceracea*	HQ234603			BM-6656
51	*Pyrenodesmiachalybaea*	KC884498	MH100779	MH100747	CBFS:JV4059
52	*Pyrenodesmiacircumalbata*	MH104923	MH100780	MH100748	Halici s.n.
53	*Pyrenodesmiaconcreticola*	KC884506	MH100781	MH100749	CBFS:JV9443
54	*Pyrenodesmiaduplicata*	HQ611272			TUR-V-7513
55	*Pyrenodesmiaerodens*	MH104927	MH100788	MH100755	Vondrak 12733 (PRA)
56	*Pyrenodesmiahaematites*	GU723420	MH100789	MH100756	Vondrak 7278 (PRA)
57	*Pyrenodesmiahaematites*	GU723421			JS280
58	*Pyrenodesmiahaematites*	MH104928			Vondrak 7278 (PRA)
59	*Pyrenodesmiahelygeoides*	MH104929	MH100790	MH100757	Frolov 1414
60	*Pyrenodesmiamicromarina*	NR_156257			CBFS:JV8199
61	*Pyrenodesmiamicromarina*		MH100791	MH100758	Vondrak 7236 (PRA)
62	*Pyrenodesmiamicromontana*	NR_158297	MH100792	MH100759	CBFS:JV9467
63	*Pyrenodesmiamicrostepposa*	NR_156260		MH100760	CBFS:JV9141
64	*Pyrenodesmiamolariformis*	KC416145	MH100793	MH100761	Nadyeina 132 (KW)
65	*Pyrenodesmiaobscurella*	MH104938		MH100762	Vondrak 7641 (PRA)
66	*Pyrenodesmiapeliophylla*	MG733135			Jason Hollinger:16476
67	*Pyrenodesmiapratensis*	MH104933	MH100795	MH100765	MIN 891605
**68**	***Pyrenodesmiarugosa***	**MW832828**	**MW832825**	**MW832904**	**BDNA-L-0001099**
69	*Pyrenodesmiatranscaspica*	MH104936	MH100799	MH100768	Vondrak 9430 (PRA)
70	*Pyrenodesmiavariabilis*	KT291466	KT291514	KT291561	Ulf Arup L07196 (LD)
71	*Shackletoniabuelliae*	KC179117			Sochting 7583
72	*Shackletoniasiphonospora*	KC179121			Sochting 7883
73	*Squamuleagalactophylla*	KC179122			Morse 10997 (LD)
74	*Squamuleakiamae*	KC179123			Kondratyuk 20480 (LD)
75	*Squamuleaparviloba*	KC179124			Wetmore 87830 (LD)
76	*Squamuleasquamosa*	MT967462			Moberg 8782 (UPS)
77	*Squamuleasquamosa*	KC179125			Karnefelt AM960105 (LD)
78	*Squamulea* ‘*squamosa*’	MT967465			Bungartz 7428 (CDS 37915)
79	*Squamuleasubsoluta*	AF353954			Arup L97072
80	*Squamuleasubsoluta*	DQ173238			Arup L97829
81	*Squamuleasubsoluta*	KJ133480			KoLRI 011067
82	‘Squamulea’ chelonia	MT967448			Bungartz 4521 (CDS 28607)
83	‘Squamulea’ chelonia	MT967451			Bungartz 9251 (CDS 46069)
84	‘Squamulea’ chelonia	MT967452			Bungartz 6146 (CDS 34358)
85	‘Squamulea’ humboldtiana	MT967439			Buck 29560 (MIN)
86	‘Squamulea’ humboldtiana	MT967440			Bungartz 4711B (CDS 56235)
87	‘Squamulea’ humboldtiana	MT967441			Bungartz 9985 (CDS 47354)
88	‘Squamulea’ oceanica	MT967445			Yánez-Ayabaca 2023 (CDS 48373)
89	‘Squamulea’ oceanica	MT967446			Bungartz 10152 (CDS 47571)
90	‘Squamulea’ oceanica	MT967447			Bungartz 9857 (CDS 47195)
91	‘*Squamulea’ osseophila*	MT967455			Aptroot 65489 (CDS 32078)
92	‘Squamulea’ phyllidizans	MT967456			Aptroot 65468 (CDS 32057)
93	‘Squamulea’ phyllidizans	MT967457			Bungartz 4710 (CDS 28808)
94	‘Squamulea’ phyllidizans	MT967458			Bungartz 4158 (CDS 28189)
95	‘Squamulea’ subsoluta	KJ133481			KoLRI 012491
96	‘*Squamulea*’ sp.	MG954160			Vondrak 18682
97	*Usnochromacarphineum*	KC179468	KC179598	KC179259	Roux s.n.
98	*Usnochromascoriophilum*	JQ301664	JQ301496	JQ301560	P. & B. v.d. Boom 38386
	**Overall**	**98**	**38**	**33**	

DNA sequences which were generated in this study, i.e. two new species, such as *Pyrenodesmiarugosa* and *Huriellaaeruginosa* are presented in bold. All others were obtained from GenBank. The species names are followed by GenBank accession numbers and voucher information. ITS, internal transcribed spacer; mtSSU, mitochondrial small subunit; LSU, large subunit; Voucher, voucher information.

## Taxonomy

### Pyrenodesmia rugosa

Taxon classificationFungiTeloschistalesTeloschistaceae

B.G. Lee & J.-S. Hur sp. nov.

C09EC14C-2C85-565C-ACE5-2E8D13954457

839184

Fig. 6

#### Diagnosis.

*Pyrenodesmiarugosa* differs from *P.micromontana* by thicker thallus (125–200 μm vs. 95–125 μm), rugose areoles (vs. flat areoles), larger apothecia (0.2–0.7 mm diam. vs. 0.2–0.4 mm diam.), shorter hymenium (60–70 μm vs. 80–100 μm), shorter hypothecium (50–55 μm vs. 80–100 μm) and narrower tip cells of paraphyses (3–4.5 μm vs. 5–6 μm).

**Figure 6.**
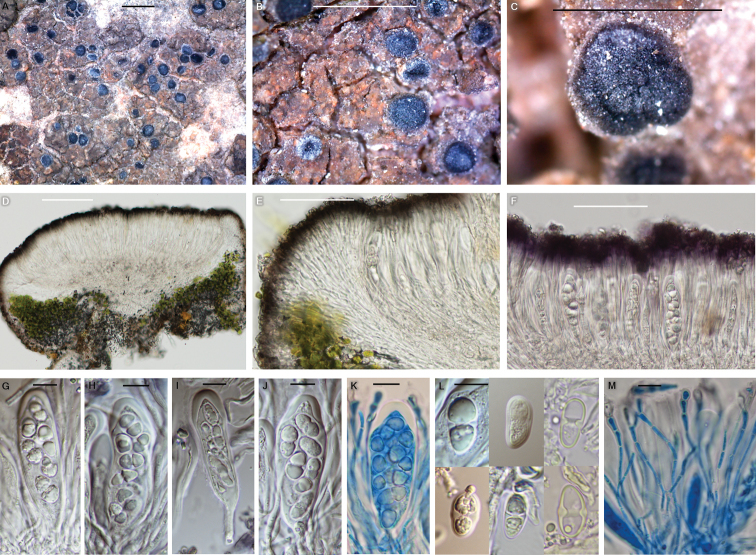
*Pyrenodesmiarugosa* (BDNA-L-0001102, holotype) in morphology **A–C** habitus and apothecia. Rugose thallus brown with orange spots and without pruina, but black apothecia often white pruinose **D–E** zeorine apothecia with well-developed parathecium. Algal layers continue to the base and underlying the hypothecium **F** epihymenium K+ purple and tiny granules not dissolving in K **G–K** asci oblong to narrowly clavate with eight spores **K** in the lactophenol cotton blue **L** ascospores simple in the beginning and developed polarilocular at maturity **M** paraphyses anastomosing in lactophenol cotton blue. Paraphysis tips slightly swollen. Scale bars: 1 mm (**A–C**); 100 μm (**D**); 50 μm (**E, F**); 10 μm (**G–M**).

#### Type.

South Korea, Gangwon Province, Gangneung, Okgye-myeon, Mt. Seokbyung (summit), 37°35.21'N, 128°53.87'E, 1,072 m alt., on calcareous rock, 17 June 2020, B.G.Lee & H.J.Lee 2020-000902, with Athalliacf.vitellinula (Nyl.) Arup, Frödén & Søchting, *Bagliettoabaldensis* (A. Massal.) Vězda, *Catillarialenticularis* (Ach.) Th. Fr. and Staurotheleaff.succedens (Rehm) Arnold (holotype: BDNA-L-0001102!); same locality, on calcareous rock, 17 June 2020, B.G.Lee & H.J.Lee 2020-000899, with Athalliacf.holocarpa (Hoffm.) Arup, Frödén & Søchting and Staurothelecf.rupifraga (A. Massal.) Arnold (paratype: BDNA-L-0001099; GenBank MW832828 for ITS, MW832825 for mtSSU and MW832804 for LSU).

Thallus saxicolous (calcicolous), crustose, mainly areolate or slightly rimose, rugose, greyish-brown to pale brown, often with orange spots, margin indeterminate or determinate when placodioid areoles are arranged around edge, vegetative propagules absent, areoles 0.4–1.0 mm diam., 125–200 μm thick; cortex hyaline with pale brown pigment layer, pale brown pigment K+ purple, 10–40 μm thick, cortical cells granular, 5–10 μm diam., with epinecral layer, 5–7 μm thick; medulla 60–110 μm thick below algal layer or inconspicuous and algal layer shown just above substrate; photobiont coccoid, cells globose to oval, 5–15 μm diam., algal layer 50–70 μm thick. Small crystals present between algal cells, not dissolving in K. Prothallus absent.

Apothecia abundant, scattered or concentrated in centre, rounded, often contiguous or even coalescent when mature, emerging on the surface of thallus, immersed or adnate, slightly constricted at the base, 0.2–0.7 mm diam. Disc flat when young and flat or concave when mature, often white pruinose, black, 200–300 μm thick; zeorine, margin persistent, slightly prominent, generally entire or rarely slightly crenulate, thalline margin paler to disc and showing brown colour, often inconspicuous due to locating below proper margin, proper margin concolorous to disc. Amphithecium present, with small crystals between algal cells, not dissolving in K, 80–130 μm wide laterally, algal layers continuous to the base and underlying the hypothecium, algal cells 5–15 μm diam., cortical layer hyaline with pale brownish pigment at periphery, 10–40 μm thick. Parathecium well-developed, hyaline, but grey with slightly brown pigment concolorous to epihymenium at periphery, 20–40 μm wide laterally and 50–90 μm wide at periphery. Epihymenium grey with slightly brown pigment, K+ purple, tiny granules abundant on surface, not dissolving in K, 5–10 μm high. Hymenium hyaline, 60–70 μm high. Hypothecium hyaline, base open and extending downwards, 50–55 μm high. Oil droplets present in upper hypothecium, but absent in hymenium. Paraphyses septate, often anastomosing, 2–2.5 μm wide, generally simple, but occasionally branched at tips, tips slightly swollen, not pigmented, 3.0–4.5 μm wide. Asci oblong to narrowly clavate, 8-spored, 52–60 × 14–18 μm (n = 5). Ascospores ellipsoid, 1-septate, polarilocular when mature or narrow septum remaining, hyaline permanently, 11–18 × 5.5–11 μm (mean = 14.1 × 7.6 μm; SD = 1.6(L), 1.0(W); L/W ratio 1.5–2.5, ratio mean = 1.9, ratio SD = 0.3; n = 105), septum 1.5–3.0 μm. Pycnidia not detected.

#### Chemistry.

Thallus K–, KC–, C–, Pd–. Epihymenium K+ purple. Hymenium I+ blue. UV–. No lichen substance was detected by TLC.

#### Distribution and ecology.

The species occurs on the calcareous rock. The species is currently known from the type collections.

#### Etymology.

The species epithet indicates the lichen’s thallus texture, rugose or wrinkled, which is the key characteristic distinguished from closely-related calcicolous species in the genus *Pyrenodesmia*.

#### Notes.

The new speices is similar to *P.micromontana*, *P.microstepposa* and *Caloplacamicromarina* Frolov, Khodos. & Vondrák in having epilithic thallus without vegetative propagules, small apothecia generally less than 0.5 mm diameter and the substrate preference to calcareous rocks. The new species differs from *P.micromontana* by thicker thallus (125–200 μm vs. 95–125 μm), rugose areoles (vs. flat areoles), larger apothecia (0.2–0.7 mm diam. vs. 0.2–0.4 mm diam.), shorter hymenium (60–70 μm vs. 80–100 μm), shorter hypothecium (50–55 μm vs. 80–100 μm) and narrower tip cells of paraphyses (3–4.5 μm vs. 5–6 μm) (Frolov et al. 2016).

The new species is different from *P.microstepposa* by darker thallus (greyish-brown to pale brown vs. ochre, grey or grey-white), rugose thallus (vs. flat thallus), thinner thallus (125–200 μm vs. 85–370 μm), smaller algal cells (5–15 μm diam. vs. 13.5–20.5 μm diam.), presence of pruina on disc (vs. absence of it), absence of oil droplets in hymenium (vs. presence of it), greyish epihymenium (vs. brownish epihymenium), wider ascospores (11–18 × 5.5–11 μm with the L/W ratio of 1.5–2.5 vs. 13.6–18.4 × 6–7.9 μm with the ratio of 1.9–2.9) (Frolov et al. 2016).

The new species is distinguished from *C.micromarina* by darker thallus (greyish-brown to pale brown vs. ochre to grey), rugose thallus (vs. flat thallus), absence of pruina on thallus (vs. presence of it), shorter hymenium (60–70 μm vs. 90–100 μm), shorter septum (1.5–3 μm vs. 2.6–3.4 μm) and the habitat preference to mountain rocks (vs. coastal rocks) (Frolov et al. 2016).

Additional specimens examined: South Korea, Gangwon Province, Okgye-myeon, Mt. Seokbyung (summit), 37°35.21'N, 128°53.87'E, 1,072 m alt., on calcareous rock, 17 June 2020, B.G.Lee & H.J.Lee 2020-000889, with *Bagliettoabaldensis*, *Catillarialenticularis*, *Fulgogasparreadecipioides* (Arup) S.Y. Kondr., M.H. Jeong, Kärnefelt, Elix, A. Thell & Hur and *Laundoniaflavovirescens* (Wulfen) S.Y. Kondr., Lőkös & Hur (BDNA-L-0001089); same locality, on calcareous rock, 17 June 2020, B.G.Lee & H.J.Lee 2020-000909, with *Bagliettoabaldensis*, *Rusavskiaelegans* (Link) S.Y. Kondr. & Kärnefelt and *Verrucarianigrescens* Pers. (BDNA-L-0001109); same locality, on calcareous rock, 17 June 2020, B.G.Lee & H.J.Lee 2020-000910, with *Bagliettoabaldensis*, *Catillarialenticularis* and *Laundoniaflavovirescens* (BDNA-L-0001110); same locality, on calcareous rock, 17 June 2020, B.G.Lee & H.J.Lee 2020-000911, with Athalliacf.vitellinula, *Bagliettoabaldensis*, *Lichenella* sp. and *Rusavskiaelegans* (BDNA-L-0001111); same locality, on calcareous rock, 17 June 2020, B.G.Lee & H.J.Lee 2020-000913, with Athalliacf.vitellinula, *Bagliettoabaldensis*, *Endocarpon* sp., *Laundoniaflavovirescens*, *Lichenella* sp. and *Rusavskiaelegans* (BDNA-L-0001113); same locality, on calcareous rock, 17 June 2020, B.G.Lee & H.J.Lee 2020-000916, with *Caloplaca* sp., *Endocarpon* sp., *Lichenella* sp. and *Rusavskiaelegans* (BDNA-L-0001116).

### Huriella aeruginosa

Taxon classificationFungiTeloschistalesTeloschistaceae

B.G. Lee & J.-S. Hur sp. nov.

3012174D-00AE-55F2-8CE1-15657EF6C8E3

839185

Fig. 7

#### Diagnosis.

*Huriellaaeruginosa* differs from ‘Squamulea’ chelonia by dark greenish-grey to grey thallus without pruina (vs. yellow orange to deep orange thallus with white pruina), gold to yellow-brown epihymenium (vs. orange epihymenium), larger ascospores (7.5–12 × 4.5–7.5 μm vs. 8–10.4 × 4.7–6.0 μm) and the chemistry (thallus K–, KC– and no substance vs. thallus K+ purple, KC± purplish and the presence of parietin, teloschistin, fallacinal, parietinic acid and emodin).

**Figure 7.**
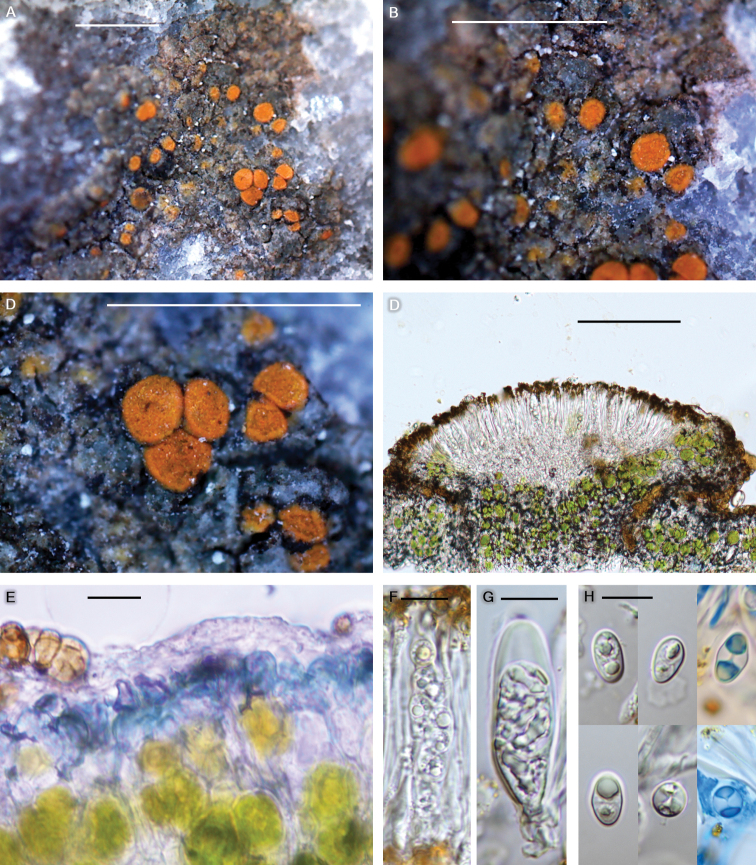
*Huriellaaeruginosa* (BDNA-L-0001072, holotype) in morphology **A–C** habitus and apothecia. Thallus dark greenish-grey to grey with no pruina. Thalline margin of apothecia concolorous to disc **D** apothecia adnate or rarely sessile. Amphithecium well-developed, but parathecium inconspicuous. **E** thallus with dark green pigment layer under cortex **F–G** clavate asci containing 8-spores **H** ascospores generally ellipsoid, but occasionally globose, developing polarilocular in both types. Two blue coloured spores in lactophenol cotton blue. Scale bars: 1 mm (**A–C**); 100 μm (**D**);10 μm (**E–H**).

#### Type.

South Korea, Gangwon Province, Gangneung, Okgye-myeon, Mt. Seokbyung (summit), 37°35.21'N, 128°53.87'E, 1,072 m alt., on calcareous rock, 17 June 2020, B.G.Lee & H.J.Lee 2020-000872, with *Bagliettoabaldensis*, *Catillarialenticularis*, *Endocarponsubramulosum* Y. Joshi & Hur, *Laundoniaflavovirescens*, *Rusavskiaelegans* and *Verrucarianigrescens* (holotype: BDNA-L-0001072!; GenBank MW832829 for ITS).

Thallus saxicolous (calcicolous), crustose, mainly areolate or slightly rimose, placodioid around edge, but without distinct lobes, thin, dark greenish-grey to grey, occasionally pale yellowish-grey when young, margin indeterminate or determinate when placodioid areoles are arranged around edge, vegetative propagules absent, areoles 0.3–0.7 mm diam., 150–200 μm thick; cortex hyaline with dark green pigment layer, 15–25 μm thick, cortical cells granular, coarsely anticlinally arranged, 5–10 μm diam., with epinecral layer, up to 5 μm thick; medulla 80–100 μm thick, below algal layer, with large crystals (materials of substrate possibly) and brown cells (dead algal cells possibly); photobiont coccoid, cells globose to oval, 5–25 μm. Small crystals in cortex, medulla and between algal cells, dissolving in K. Prothallus absent.

Apothecia abundant, scattered and not concentrated in centre, rounded, often contiguous when mature, emerging on the surface of thallus, immersed, adnate or rarely sessile, constricted at the base, 0.2–0.4 mm diam. Disc flat when young and flat or slightly convex when mature, not pruinose, orange from the beginning, 110–230 μm thick; margin persistent, even to disc or slightly prominent, generally entire or slightly crenulate, thalline margin concolorous to disc, proper margin inconspicuous. Amphithecium well-developed, with small crystals between algal cells, dissolving in K, 50–55 μm wide laterally, algal layers continuous to the base or solitarily remaining in amphithecium, algal cells 5–25 μm diam., cortical layer hyaline with gold to yellow-brown pigment concolorous to epihymenium at periphery, 15–20 μm thick. Parathecium inconspicuous, hyaline but gold to yellow-brown at periphery, ca. 10 μm wide laterally and ca. 20 μm wide at periphery. Epihymenium gold to yellow-brown, granular, pigment K+ wine red and dissolved, 10–20 μm high. Hymenium hyaline, 45–55 μm high. Hypothecium hyaline, 35–45 μm high. Oil droplets present, small, along paraphyses and more in the base of hymenium and hypothecium. Paraphyses septate, anastomosing, 2–3 μm wide, simple or branched at tips, tips swollen or slightly swollen, not pigmented, 3.5–5.5 μm wide. Asci clavate, 8-spored, 35–48 × 14–17 μm (n = 5). Ascospores generally ellipsoid, occasionally globose, 1-septate, polarilocular or narrow septum remaining, hyaline permanently, 7.5–12 × 4.5–7.5 μm (mean = 9.9 × 5.7 μm; SD = 0.9(L), 0.6(W); L/W ratio 1.2–2.3, ratio mean = 1.8, ratio SD = 0.2; n = 104), globose spores 7.5–9 × 7.0–9.2 μm (mean = 8.0 × 7.7 μm; SD = 0.8(L), 0.9(W); L/W ratio 1.0–1.1, ratio mean = 1.0, ratio SD = 0.1; n = 11). Pycnidia not detected.

#### Chemistry.

Thallus K–, KC–, C–, Pd–. Apothecia K+ wine red. Epihymenium K+ wine red. Epihymenium and hymenium I+ blue. UV–. No lichen substance was detected by TLC.

#### Distribution and ecology.

The species occurs on the calcareous rock. The species is currently known from the type collection.

#### Etymology.

The species epithet indicates the lichen’s thallus colour, dark green, which is the key characteristic distinguished from all the species in the genus *Huriella*.

#### Notes.

The morphological classification of the new species is not clear between *Huriella* and *Squamulea* because the new species has some characteristics for the former genus and others for the latter, i.e. the new species represents mainly areolate thallus without lobed margin and smaller apothecia for the former, whilst showing some squamulose thallus and wider ascospores for the latter (Table 2). The molecular results concluded the new species classification into the former genus, *Huriella*.

**Table 2.** Comparison of the new species with two type species in *Huriella* and *Squamulea*.

Species	Huriellaaeruginosa	Huriellaloekoesiana	Squamuleasubsoluta
Thallus	mainly areolate, rimose or placodioid around edge, but without lobes	areolate (not squamulose)	squamulose, areolate or subsquamulose, margin slightly lobed
Apothecia (mm in diam.)	0.2–0.4	0.2–0.4(–0.5)	0.1–0.6
Ascospores (μm)	7.5–12 × 4.5–7.5	(8.5–)9–11(–12) × (4.5)5–6	9.5–12.5 × 5.5–7
Molecular phylogeny	Huriella	Huriella	Squamulea
Reference	–	Kondratyuk et al. 2017b	Nash III TH et al. 2007; Arup et al. 2013

The new species is unique with the key characteristics of green pigmented thallus (with a distinct green layer in a section) and the substrate preference to calcareous rocks amongst all *Huriella* species.

The new species is similar to ‘Squamulea’ chelonia, *Squamuleagalactophylla*,‘Squamulea’ humboldtiana, *S.parviloba* and *S.subsoluta* in the substrate preference to calcareous rocks. However, the new species is different from ‘Squamulea’ chelonia by dark greenish-grey to grey thallus without pruina (vs. yellow orange to deep orange thallus with white pruina), gold to yellow-brown epihymenium (vs. orange epihymenium), larger ascospores (7.5–12 × 4.5–7.5 μm vs. 8–10.4 × 4.7–6.0 μm) and the chemistry (thallus K–, KC– and no substance vs. thallus K+ purple, KC± purplish and the presence of parietin, teloschistin, fallacinal, parietinic acid and emodin) (Bungartz et al. 2020).

The new species differs from *S.galactophylla* by thallus colour (dark greenish-grey to grey vs. dirty white to yellowish-brown), flat to convex disc (vs. flat disc only), yellowish-orange apothecia (vs. cinnamon-brown apothecia), smaller ascospores (7.5–12 × 4.5–7.5 μm vs. 10–15 × 5–7 μm) (Fink 1935; Arup 2013).

The new species is distinguished from‘Squamulea’ humboldtiana by dark greenish-grey to grey thallus without pruina (vs. yellow-orange to deep orange thallus with pruina), absence of prothallus (vs. presence of prothallus), larger ascospores (7.5–12 × 4.5–7.5 μm vs. 8.1–9.9 × 4.8–5.9 μm) and the chemistry (thallus K–, KC– and no substance vs. thallus K+ purple, KC± purplish and the presence of parietin, teloschistin, fallacinal, parietinic acid and emodin) (Bungartz et al. 2020).

The new species differs from *S.parviloba* by dark greenish-grey to grey thallus (vs. yellow-orange to orange thallus), absence of lobes (vs. short narrow elongated lobes around edge), convex and yellow-orange disc (vs. flat and deep orange disc), smaller ascospores (7.5–12 × 4.5–7.5 μm vs. 11–14 × 5.5–7 μm) and the chemistry (thallus K– vs. thallus K+ red) (Wetmore 2003; Nash III TH et al. 2007).

The new species is different from *S.subsolutaby* dark greenish-grey to grey thallus (vs. yellow-orange, orange to reddish-orange thallus), absence of prothallus (vs. black prothallus), flat to convex, yellow-orange apothecia (vs. flat to concave, deep orange apothecia) and the chemistry (thallus K– and no substance vs. thallus K+ red, the presence of parietin, fallacinal, emodin and teloschistin) (Wetmore 2003; Nash III TH et al. 2007).

The most distinctive characteristic of the new species is the thallus colour, i.e. dark greenish-grey to grey, which is different from all comparable calcicolous species in the genus *Squamulea*.

### Key to the species of *Huriella* and *Squamulea* (20 taxa)

Although some species of *Huriella* have distinct characteristics, different from *Squamulea*, such as mainly areolate and non-squamulose thallus without lobes at margin, smaller apothecia and narrower ascospores (Kondratyuk et al. 2017b), those morphological taxonomic keys do not clearly separate the two genera concerning all known species in the genera. The morphological characteristics are assumingly based on the comparison between type species of the comparable genera, but several species do not correspond to the characteristics (e.g. *Huriellaaeruginosa*, *H.flakusii* Wilk and *H.salyangiana* S.Y. Kondr. & Hur with squamulose thalli), although those species are classified in the genus *Huriella* in molecular phylogeny. Such a discrepancy between morphology and molecular phylogeny occur in *Squamuleasquamosa* (B. de Lesd.) Arup, Søchting & Frödén and *S.subsoluta* as well. Both species are considered conspecific in morphology. Both species are very similar in morphology and ecology occurring together on the same rock. Whereas the only difference between them is that the former has a thalline margin and it is lacking in the latter (Nash III TH et al. 2007), the latter representing a permanent thalline margin from the Galapagos Islands as well (Bungartz et al. 2020). However, the two species are separated and located distant from each other in molecular results of this study (Fig. 5). Nevertheless, those are still considered conspecific in the key below as a taxonomic key is based mainly on ecology, morphology and chemistry. The genera *Huriella* and *Squamulea* should be more studied in the future and here a preliminary key is updated from previous taxonomic keys of Wetmore (2003) and Bungartz et al. (2020).

**Table:** 

1	Not directly on rock, but on lichen or bone	**2**
–	On rock	**4**
2	On lichen (*Aspicilia*) living on rock	***Squamuleanesodes***
–	On bone	**3**
3	Thallus generally areolate, without blastidia, not pruinose	‘ ***Squamulea*’ *osseophila***
–	Thallus generally (sub)squamulose, blastidia abundant, not pruinose or faintly orange pruinose on thallus	‘ ***Squamulea*’ *phyllidizans***
4	On calcareous rocks	**5**
–	On siliceous rocks	**10**
5	Thallus pruinose	**6**
–	Thallus not pruinose	**7**
6	Thallus angular, areolate to subsquamulose, prothallus absent	‘ ***Squamulea*’ *chelonia***
–	Thallus areolate or bullate, prothallus black when present	‘ ***Squamulea*’ *humboldtiana***
7	Thallus whitish, greyish or greenish	**8**
–	Thallus yellow-orange to orange	**9**
8	Thallus dirty whitish, disc cinnamon-brown	***Squamuleagalactophylla***
–	Thallus dark greenish-grey to grey, disc orange	***Huriellaaeruginosa***
9	Areole margins with small lobules	***Squamuleaparviloba***
–	Areole margins without lobules	***Squamuleasquamosa* (*S.subsoluta*)**
10	With blastidia or soredia	**11**
–	Without blastidia or soredia	**13**
11	Thallus brownish-orange, apothecia rare, disc reddish to reddish-brown, ascospores 11–16 × 6–8 μm, isthmus 1–3 μm	***Squamuleakiamae***
–	Thallus yellowish-orange to deep orange, apothecia common, disc concolorous to thallus or slightly deeper, ascospores 8.4–13.3 × 5–7.1 μm, isthmus 2.5–4.6 μm	**12**
12	Blastidia abundant, sometimes faintly orange pruinose on thallus, but not pruinose on disc	‘ ***Squamulea*’ *phyllidizans***
–	Soredia rarely present, rarely white pruinose on disc, but not pruinose on thallus	***Squamuleasquamosa* (*S.subsoluta*)**
13	Thallus areolate to (sub)squamulose	**14**
–	Thallus areolate or bullate, but not squamulose	**21**
14	Prothallus distinctly blackened	‘ ***Squamulea*’ *oceanica***
–	Prothallus absent	**15**
15	Disc brownish to reddish or blackish	**16**
–	Disc orangish	**19**
16	Thallus orange, disc reddish, ascospores 11–14.2 × 5.9–7.5 μm	***Huriellaflakusii***
–	Thallus brownish, disc pale brown, brownish-orange to blackish-brown	**17**
17	Disc dark brown-orange to black-brown, hypothecium 20–30 μm high, ascospores 7–9 × 4.5–6.5 μm	***Huriellasalyangiana***
–	Disc pale brown to brownish-orange, hypothecium 50–150 **μm** high, ascospores 9–13 × 4.5–6 μm	**18**
18	Disc 0.4–0.9 mm diam., hypothecium 50–100 μm high, ascospores 9–13 × 5–6 μm	***Squamuleacoreana***
–	Disc 0.2–0.4 mm diam., hypothecium 100–150 μm high, ascospores 10–10.5 × 4.5–6 μm	***Squamuleauttarkashiana***
19	Areole margins with small lobules	***Squamuleaparviloba***
–	Areole margins without lobules	**20**
20	Ascospores 8–10.4 × 4.7–6 μm, isthmus 2.1–3.3, not pruinose on disc	‘ ***Squamulea*’ *chelonia***
–	Ascospores 8.4–13.3 × 5.2–7 μm, isthmus 2.5–4 μm, rarely pruinose on disc	***Squamuleasquamosa* (*S.subsoluta*)**
21	Thallus yellow-orange to deep orange, prothallus black when present, ascospores 8.1–9.9 × 4.8–5.9 μm, isthmus 2.7–3.2 μm	‘ ***Squamulea*’ *humboldtiana***
–	Thallus yellow-brownish or yellow-greenish, prothallus absent, ascospores 9–15 × 5–8 μm, isthmus 2–5 μm	**22**
22	Apothecia 0.2–0.3 mm diam., disc dull brown, dull yellow to bright yellow	**23**
–	Apothecia 0.3–1 mm diam., disc orange, brownish-yellow to reddish-orange	**24**
23	Disc dull yellow to bright yellow, hymenium 50–60 μm high, hypothecium 20–30 μm high, ascospores 9–11 × 5–6 μm, isthmus 4–5 μm	***Huriellaloekoesiana***
–	Disc dull brown, hymenium 80–100 μm high, hypothecium 80–110 μm high, ascospores 13–14.5 × 7–8 μm, isthmus 3–4 μm	***Huriellaupretiana***
24	On mountain, thallus yellow-brown, disc orange, isthmus 3–4 μm	***Squmuleamicromera***
–	On coast, thallus dull green-yellow to yellow-brown, disc orange to red-orange, isthmus 2–3 μm	***Huriellapohangensis***

## Supplementary Material

XML Treatment for Pyrenodesmia rugosa

XML Treatment for Huriella aeruginosa
